# Emergence of a neopelagic community through the establishment of coastal species on the high seas

**DOI:** 10.1038/s41467-021-27188-6

**Published:** 2021-12-02

**Authors:** Linsey E. Haram, James T. Carlton, Luca Centurioni, Mary Crowley, Jan Hafner, Nikolai Maximenko, Cathryn Clarke Murray, Andrey Y. Shcherbina, Verena Hormann, Cynthia Wright, Gregory M. Ruiz

**Affiliations:** 1 Smithsonian Environmental Research Center, Edgewater, MD USA; 2 Ocean and Coastal Studies Program of Williams College and Mystic Seaport Museum, Mystic, CT USA; 3 Lagrangian Drifter Laboratory, Scripps Institution of Oceanography, University of California San Diego, La Jolla, CA USA; 4 Ocean Voyages Institute, Sausalito, CA USA; 5 International Pacific Research Center, School of Ocean & Earth Science & Technology, University of Hawaii at Manoa, Honolulu, HI USA; 6 Institute of Ocean Sciences, Fisheries and Oceans Canada, Sidney, BC Canada; 7 Applied Physics Laboratory, University of Washington, Seattle, WA USA

**Keywords:** Biooceanography, Community ecology, Marine biology

## Abstract

Discoveries of persistent coastal species in the open ocean shift our understanding of biogeographic barriers. Floating plastic debris from pollution now supports a novel sea surface community composed of coastal and oceanic species at sea that might portend significant ecological shifts in the marine environment.

The ubiquitous spread and increase of plastic pollution have galvanized international attention and attracted rapidly growing research, focused on diverse effects. In the coming decades, the effects of plastic pollution in the marine environment are expected to expand, with plastic production and waste predicted to exponentially increase, reaching an estimated total of 25,000 million metric tons of waste generation by 2050^1^. Here, we discuss an additional and overlooked cascading consequence of plastic pollution, triggered by the introduction of an immense floating plastic habitat in the open ocean^2,3^—the unpredicted establishment of coastal species in high seas ocean gyres.

We define this emergent novel ecosystem as the neopelagic community (Fig. 1). Although the transport of coastal species across oceans and along coasts on floating debris, also known as ocean rafting, has long been known to occur on natural rafts, including seeds, trees, seaweeds and pumice, past documented occurrences were assumed to be ephemeral^4^. The extent and frequency of coastal species on rafts in the ocean were likely historically constrained, due to the biodegradable nature and, therefore, relatively short longevity of natural materials^5,6^, as well as a likely limited and highly episodic raft supply. Nevertheless, the presumed ability of coastal species to survive ocean transits has been a fundamental tenet of island biogeography and thought to explain the presence of continentally derived species on oceanic islands^7^. Thus, until now, consideration of rafting of coastal species focused primarily on transient passage through the ocean rather than long-term residency and colonization of the open ocean. With globalization during the Anthropocene, biogeographic barriers historically imposed by oceans and continents are quickly becoming obsolete—socially, economically, and now ecologically. The global increase in plastic pollution is an unexpected example of this effect, where plastic rafts create a more permanent opportunity for coastal species to transit ocean basins and a long-term enduring habitat to colonize in the open ocean.

**Fig. 1:**
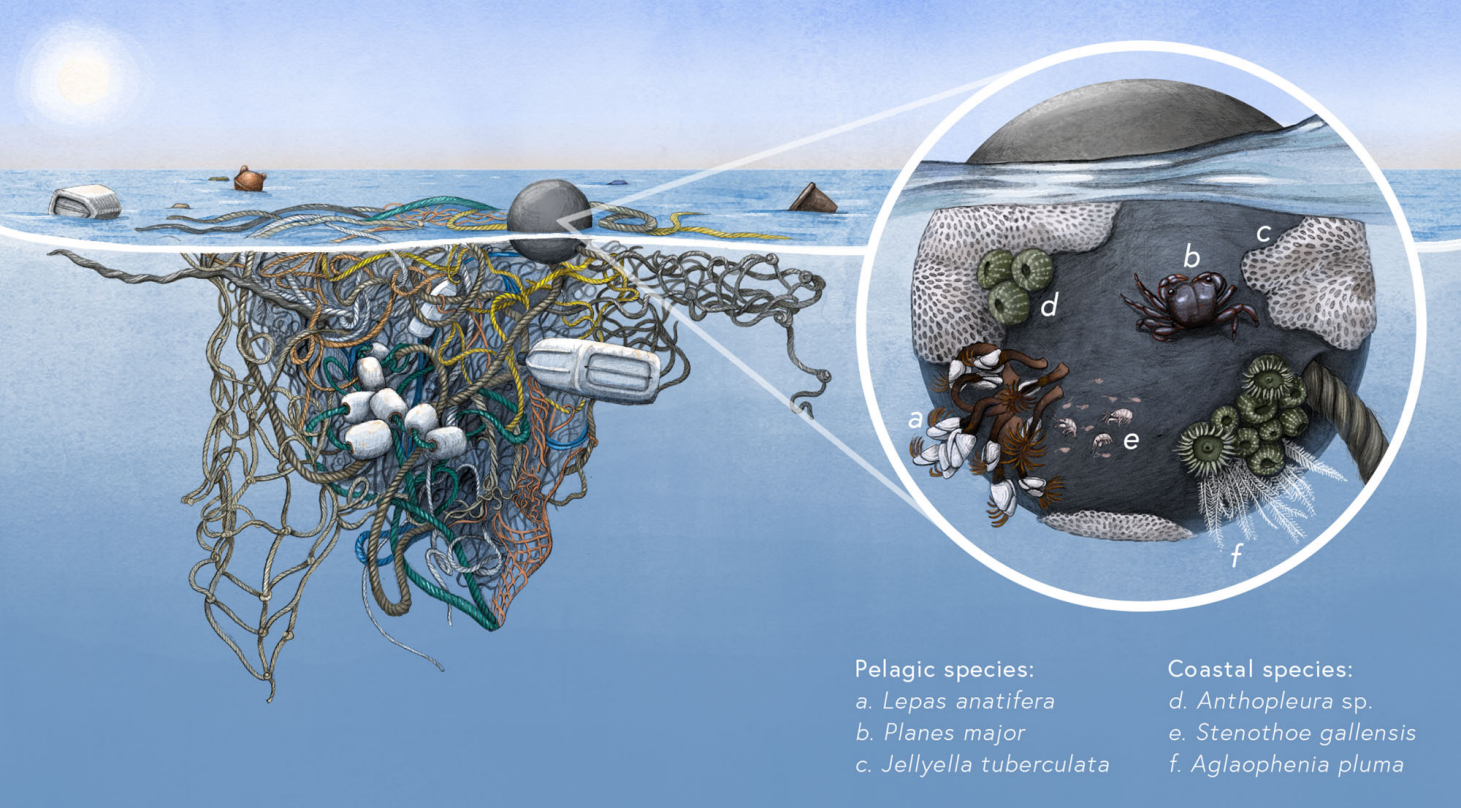
Neopelagic community rafting on floating plastic debris in ocean surface waters. Neopelagic communities are composed of pelagic species, evolved to live on floating marine substrates and marine animals, and coastal species, once assumed incapable of surviving long periods of time on the high seas. The emergence of a persistent neopelagic community in the open ocean is due to the vast supply of durable and highly buoyant plastic pollution as suitable habitat for both pelagic and coastal rafting species. Examples of pelagic rafting species are: **a** gooseneck barnacle *Lepas anatifera*, **b** flotsam crab *Planes major*, and **c** bryozoan *Jellyella tuberculata*. Examples of coastal rafting species commonly found on floating plastic debris on the high seas include: **d** podded hydroid *Aglaophenia pluma*, **e** Asian anemones *Anthopleura* sp., and **f** amphipod *Stenothoe gallensis*. Illustrated by © 2021 Alex Boersma.

## Tsunami exposes the role of rafts in transoceanic dispersal

**Fig. 2:**
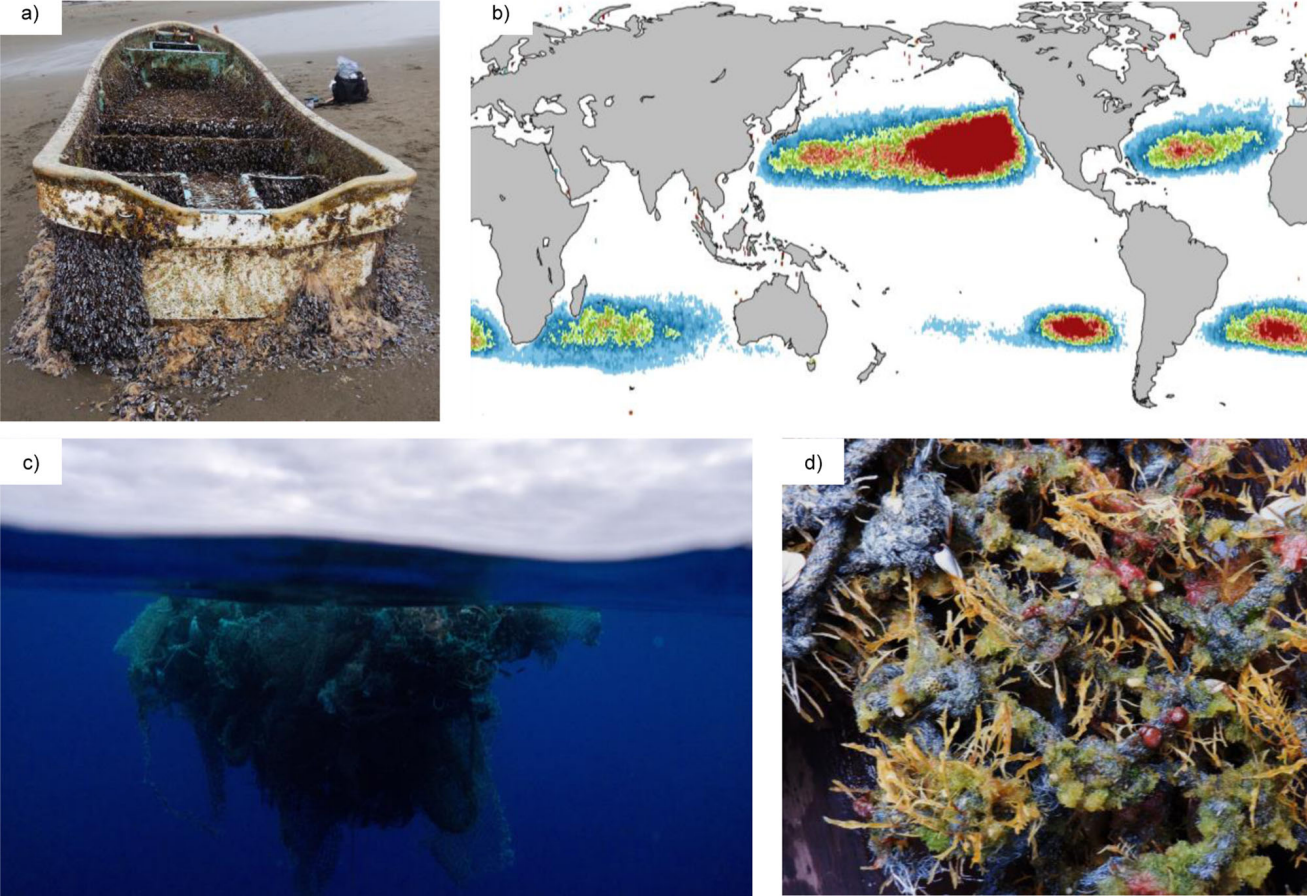
Floating plastic debris may be an underestimated vector of coastal species transport on the high seas. **a** Vessel identified as Japanese tsunami debris landed in southern Oregon on May 13, 2020 with ten living invertebrate species aboard, all of which are representative of the coastal Northwest Pacific Ocean, including mussel *Musculus cupreus*, bryozoan *Bugula tsunamiensis*, and isopod *Ianiropsis serricaudis*. All coastal species attached undergo direct development or asexual reproduction. **b** Areas of floating plastic debris accumulation simulated with numerical drift model^12^, showing plastic debris concentrating in the world’s five major gyres; red to blue depicts relative high to low concentrations of debris. **c** Mass of derelict fishing gear, composed of plastic nets, ropes, and buoys, in the North Pacific Subtropical Gyre; fishing nets have not been recognized as Japanese tsunami debris. **d** Neopelagic community attached to derelict fishing net recovered from the North Pacific Subtropical Gyre. Coastal taxa attached include Asian anemone *Anthopleura* sp., as well as the hydroid *Aglaophenia pluma*, and vase sponge *Sycon* sp. Pelagic taxa include gooseneck barnacle *Lepas anatifera*. Debris depicted in **a** were photographed by Nancy Treneman. Those in images **c** and **d** were photographed by Ocean Voyages Institute in the North Pacific Subtropical Gyre during their 2020 expedition.

Our understanding of ocean rafting unequivocally shifted following the devastating East Japan Earthquake and Tsunami of 2011 and the subsequent debris field ejected into the North Pacific Ocean. Hundreds of coastal Japanese marine species were found alive on the debris that landed on the shores of the North American Pacific coast and the Hawaiian Islands, having traveled over 6000 km across the Pacific Ocean—the largest ocean rafting event known in scientific literature to date (Fig. 2a)^8,9^. The actual scale of this event was no doubt much greater, given the estimated diversity of coastal species on detected debris objects and the fact that many objects and their epiplastic biota went undetected^8,9^. Not only did these coastal organisms survive and apparently grow for years at sea, but several were found to have reproduced on tsunami debris in the open ocean^9^. This discovery demonstrated that anthropogenic debris, which was largely composed of floating plastics, provided long-lived, habitable rafts and exceeded our expectations of coastal species survival at sea.

Much of the debris field from the East Japan Tsunami was predicted to enter the North Pacific Subtropical Gyre, an area of convergence in the eastern Pacific Ocean that acts as a reservoir of floating plastic from sources surrounding the North Pacific. In fact, the North Pacific Subtropical Gyre is estimated to hold the greatest concentration of floating plastic of all ocean gyres^3^ (Fig. 2b). Our new observations in this gyre reveal that coastal species are not only present but are common on floating plastic debris, including objects that have been newly colonized at sea and are not from coastal sources, such as derelict fishing gear lost on the high seas (Fig. 2c, d). These observations, coupled with prior at sea records, reveal a picture of persistent coastal marine biodiversity on the high seas, sustained by plastic pollution and altering the long-held assumption that rafting coastal species reflected temporary ocean passage^9,10^.

## The neopelagic community

A shift in the composition of open ocean invertebrate communities appears to be underway with increasing diversity of coastal species on extensive floating plastic rafts that can persist for years at the ocean’s surface and that are advected thousands of kilometers by ocean currents, direct waves, and wind forcing. Historically, invertebrate communities in open ocean surface waters were composed largely of oceanic neuston. Neuston are those organisms floating or swimming at or just below the ocean surface, many of which have evolved unique mechanisms to survive at the sea-air interface^11^. From blowing bubble floats (the bubble snail *Janthina* spp.) to the growth of air sacs that catch the wind (the hydrozoan *Velella velella*), to settlement on floating logs and seaweed or marine animals (the barnacles *Lepas* spp. and crabs *Planes* spp. and *Plagusia* spp) (Fig. 1), oceanic neuston are adapted to reproduce and thrive in the open ocean^11^.

We introduce the term “neopelagic” for this new ocean surface community, which is now composed of both oceanic neuston and rafting coastal species. The emergence of the neopelagic community is likely recent, due in large part to the accumulation of long-lived plastic rafts, which have been increasing rapidly since the middle of the 20th century^2,3^. The introduction of durable, exceptionally buoyant substrate to the open ocean provides habitat for coastal (benthic) species that was previously absent or sparse. A vast area of the ocean now appears to be suitable habitat for a wide diversity of species previously restricted to coastal waters. This may be particularly true in subtropical gyre systems, where floating plastics accumulate over time (Fig. 2b)^12,13^, with possible residence times of years to decades or longer.

## An ocean invaded

The persistence of coastal species at sea presents a paradigm shift in our understanding of marine biogeography in several respects. First, the open ocean has long been considered a physical and biological barrier for dispersal of most coastal marine species, creating geographic boundaries and limiting distributions^14^. This situation no longer appears to be the case, as suitable habitat now exists in the open ocean and coastal organisms can both survive at sea for years and reproduce, leading to self-sustaining coastal communities on the high seas. Second, persistence of coastal organisms in the open ocean creates the possibility of stepping-stone dispersal; rather than plastic rafts as an ephemeral vector of coastal species from one coastal point to another across waterbodies^15^, resident coastal species in the high seas might act as a steady source of propagules both to new plastic debris moving through the gyre and to coasts via rafts.

## A sea of questions

Much remains to be learned across disciplines about the neopelagic community and ecosystem. That coastal species can survive for years in the open ocean environment has changed our prior understanding of the availability of trophic resources and of a conducive physiochemical environment to support coastal species in open ocean environments, which were previously considered inhospitable for long-term survival of coastal biota.

### Colonization and persistence

At present, we have limited understanding of the ecology of neopelagic communities. Basic questions remain unanswered, such as what is the extent of the biodiversity of coastal species persisting at sea and how often do coastal species co-occur with neustonic species on plastic rafts? Raft characteristics are known to affect neopelagic community structure, with species diversity increasing with plastic raft surface area^9,10^, but research is needed to investigate how raft characteristics shape the ecological interactions between coastal and pelagic species. Perhaps most fundamentally, we need to know to what extent neopelagic communities self-sustain or require continued input of rafts, propagules, and gene flow from coastlines. For these communities to self-sustain, coastal species traits and life histories, the physical environment, and trophic resources must align for survival, successful reproduction, and population persistence. Understanding what trophic resources coastal species utilize in the open ocean as well as the ecological roles that they play in neopelagic communities and oceanic ecosystems is crucial to understanding the impact of permanent communities of coastal species on the open ocean.

### Biogeography

The motion of floating plastic rafts is integral to future research on dynamics of coastal biota at sea since the physical oceanic environment shapes neopelagic communities. Origin might constrain neopelagic community development and composition. For example, a plastic buoy that comes loose from an offshore aquaculture facility, which is heavily fouled with coastal species upon departure, might undergo very different community succession dynamics than a plastic water bottle that falls overboard mid-ocean and is newly colonized by both neustonic and coastal species. How these objects are transported on ocean currents through space and time and the abiotic conditions encountered will further affect the neopelagic community associated with them.

In addition to transport, aggregation of floating plastic rafts in the open ocean, and specifically in gyres where plastics can remain for years, might have important implications for recruitment and gene flow of coastal species. Differences in physical oceanic features and sources of plastics among ocean regions might further contribute to a complex biogeography of neopelagic communities. Many factors could influence the biogeography of these novel communities, including the scale of plastic input and their residence times, spatial and temporal patterns of productivity, temperature, and other environmental variables. An important early step is to determine whether neopelagic communities like those found in the North Pacific form in other oceans, and if so, to what extent these communities differ among ocean basins.

### Biological invasions

Understanding the ecology and biogeography of the neopelagic communities on floating plastics will provide essential insights about the role of plastics as vectors of non-native species. The persistence of coastal species on plastic debris might increase the potential for successful transoceanic dispersal of coastal species to new continents by increasing the duration and distance of dispersal than would be possible otherwise. Additionally, colonization of plastic debris at sea by coastal species suggests that the continued expansion of the plastisphere creates a novel source pool of non-native species on the high seas. Thus, the increase of plastic inputs to the global ocean, when combined with discovery of the neopelagic community, points to an underestimation of floating plastics as vectors of transoceanic invasive species dispersal and introductions.

## Implications and repercussions

Plastic production, and plastic rafts, have increased exponentially since the 1950s^15^. Yet, the addition of a novel permanent or semi-permanent coastal biota component in the open ocean appears to have been overlooked. This oversight might be due to the relatively low amounts of plastic marine debris until recent decades, along with relatively few formal studies over the past 50 years on the rafting biology of high seas debris. Greater frequency and amounts of plastics on land, coupled with climate change-induced increases in coastal storm frequency ejecting more plastics into the ocean, will provide both more rafting material and coastal species inoculations, increasing the prevalence of the neopelagic community. As a result, rafting events that were rare in the past could alter ocean ecosystems and change invasion dynamics on a global scale, furthering the urgent need to address the diverse and growing effects of plastic pollution on land and sea.
